# Supplementary material for: A survival model for course-course interactions in a Massive Open Online Course platform
Source: PLoS One. 2021 Jan 22;16(1):e0245718. doi: 10.1371/journal.pone.0245718 (PMC7822273; doi:10.1371/journal.pone.0245718)
Supplement: S1 File — (DOCX) [file pone.0245718.s001.docx]

A survival model for course-course interactions in a Massive Open Online Course platform

Edwin H. Wintermute, Matthieu Cisel & Ariel B. Lindner

Corresponding Author: Edwin H. Wintermute

Email: ehwintermute@gmail.com

This PDF file Includes

**Supporting Text**

[Definition and Characterization of Burst Registration Events 2](#_Toc47956927)

[Detailed Derivation of the *βEND* Model 4](#_Toc47956928)

[Parameter Fitting for the *βEND* Model 6](#_Toc47956929)

[Derivation and Parameter Optimization for the Logistic Model 8](#_Toc47956930)

[Regression Results for the *βEND* Model 9](#_Toc47956931)

**Supporting Figures S1 to S3**

**Supporting Tables S1 to S4**

**References for SI Citations**

Definition and Characterization of Burst Registration Events

|  | Fig S1. User registrations often come in bursts. Each row depicts the registration activity of a representative user. Blue dots indicate registration events. Dots were stacked vertically to indicate multiple registrations on the same day. |
| --- | --- |

Figure S1 depicts registration events over time for representative users. Registrations were not uniformly distributed across the study period, but tended to cluster in time. We sought to quantify this pattern and assign individual user registrations to well defined registration burst events.

The large majority of re-registrations occurred within a few hours of a previous registration event. A significant number users also returned to the platform after a delay of weeks or months. However, re-registration delays of intermediate periods, from 2-24 hours, were relatively rare. The bi-modal nature of the delay distribution suggests that most registration events can be assigned to short-term bursts of activity that are separated by relatively long periods of inactivity.

Registration burst events were defined using agglomerative clustering in MATLAB. A hierarchical cluster tree was generated using the time between registration events as a distance metric and a nearest-neighbor linkage function. Bursts were separated from the cluster tree using a delay time cutoff of 8 hours. Varying the chosen cutoff time from 2-24 hours had little effect (<1%) on the total number of bursts defined (Fig. S2).

|  | Fig S2. Definition of registration burst events is insensitive to the selected clustering threshold. User registrations were assigned to registration burst events using an agglomerative clustering algorithm with a varying burst separation threshold. For each threshold, the total number of defined burst events is indicated. (A) The total burst count changes slowly with respect to the burst separation threshold in the vicinity of one day. (B) Varying the burst separation threshold between 2 hours and 1 day changed the total number of defined burst events by less than 1%. This indicates that the assignment of registrations to burst events is robust to the chosen clustering threshold. A clustering threshold of 6 hours was selected for further analysis. |
| --- | --- |

The assignment of registrations to burst events created two new metrics. Each user is associated with a burst number, defined as the total number of burst events recorded for that user during the study period. Each burst is associated with a burst size, the total number of courses registered during the event. We characterized the distribution of these quantities among users of the FUN platform.

Both burst size and burst number were found to follow heavy-tailed distributions that were well described by a modified power law (Fig. S3). Power law distributions are commonly found in internet user activity (1). In this case, deviation from a strict power law may be explained by the fact that the total number of available courses, and therefore the maximum number of registrations, is bounded at a maximum of 140.

The heavy-tailed character of these distributions indicates that a relatively small number of super-users are responsible for an outsized portion of registration activity. For example, 70% of FUN users were associated with only a single registration burst event. Only 15% of users were associated with 3 or more registration bursts, yet these users were responsible for 63% of the total recorded bursts. Similarly, only 12% of registration bursts were of size 3 or more, yet these bursts accounted for 35% of total recorded registrations.

The *βEND* model weights each registration event equally, regardless of the total registration activity of the associated user. Super-users may therefore have a significant influence on the global model behavior.

|  |
| --- |
| Fig S3. Burst metrics follow a modified power law. (A) Frequency distributions and model parameters for the burst number, defined as the total number of registration burst events recorded for each user. (B) Frequency distributions and model parameters for the burst size, defined as the number of course registrations recorded for each burst event. Neither distribution was well described by a strict power law but both could be fit with an additional term producing a curved power law. Best-fit parameter values are indicated for each model as well as 95% confidence intervals. |

Detailed Derivation of the *βEND* Model

| $\hat{t}$ | The pseudo-time invested by a user in a course. |
| --- | --- |
| $\hat{T}$ | The pseudo-time at which a given user withdraws from a course. |
| *β* | A Weibull shape parameter. |
| *E* | The engagement level of a user. |
| *N* | The number of courses registered in a single burst. |
| *D* | The difficulty of a course; the minimum pseudo-time investment required to certify. |
| $P\left( \hat{t} \right)$ | A probability density function. |
| $F\left( \hat{t} \right)$ | A cumulative distribution function. |
| $P\left( \hat{t} \right)$ | A survival function. |
| $S_{W}\left( \hat{t} \right)$ | A Weibull survival function. |
| $H\left( \hat{t} \right)$ | A hazard rate function. |
| $H_{W}\left( \hat{t} \right)$ | A Weibull hazard rate function. |
| Table S1. Summary of notation used in construction of the βEND model. | |

The personal investment of a user in a course is expressed in units of pseudo-time, $\hat{t}$, which may be considered as the true quantity of time spent with an unknown scaling factor to reflect personal productivity. A user may withdraw from a course after investing any amount of pseudo-time, following a probability density function $P\left( \hat{t} \right)$. User withdrawal is a passive event and not associated with any recorded action on the MOOC platform.

Following reliability models of aging, we define a survival function, $S\left( \hat{t} \right)$, as the probability that the withdrawal time for a given user, $\hat{T}$, is greater than $\hat{t}$. The survival function is simply $\begin{matrix} 1 \end{matrix}-F\left( \hat{t} \right)$ where $F\left( \hat{t} \right)$ is the cumulative distribution function of $P\left( \hat{t} \right)$.

$S\left( \hat{t} \right)=P\left( \hat{T}>\hat{t} \right)=1-P\left( \hat{T}<\hat{t} \right)=1-F\left( \hat{t} \right)$ (1)

The hazard rate, $H\left( \hat{t} \right)$, is the instantaneous relative risk that a student will withdraw from a course. It represents the withdrawal rate at $\hat{t}$ conditioned on a user having persisted for at least $\hat{t}$ units of time in the course. The hazard rate is obtained from the survival function as follows.

$H\left( \hat{t} \right)=\frac{dS}{d\hat{t}}\cdot\frac{1}{S}=\frac{-dlog\left( S \right)}{d\hat{t}}$ (2)

In the Weibull model for aging systems, the hazard rate changes with time as a power function.

$H_{W}\left( \hat{t} \right)=\frac{\beta}{E^{\beta}}\hat{t}^{\left( \beta-1 \right)}$ (3)

The term *β* is the shape parameter for the Weibull distribution. The values of *E*, $\hat{t}$, and *β* are all constrained to be positive. Note that for $\beta=1$ the hazard rate is constant in time and the Weibull model reduces to an exponential model. For $\beta>1$ the hazard rate increases with time and the system is said to experience aging. For $\beta<1$ the hazard rate slows with time. In our context, this means that the chance that a user will withdraw from a course decreases as the user invests more time.

From the Weibull hazard rate *H_W_* we obtain the Weibull survivor function *S_W_*

$S_{W}\left( \hat{t} \right)=exp\left[ -\int_{0}^{\hat{t}} H_{W}\left( x \right)dx \right]=exp\left[ -\left( \frac{\hat{t}}{E} \right)^{\beta} \right]$ (4)

The difficulty of a course, *D*, is defined as the minimum time investment required to obtain a certificate. Users who disengage from a course at any time prior to *D* will not certify it. The probability of obtaining a certificate is therefore calculated as the survival function taken at $\hat{t}=D$.

$P\left( \hat{T}>D \right)=exp\left[ -\left( \frac{D}{E} \right)^{\beta} \right]$ (5)

Finally we account for the fact that a user may have registered for N courses simultaneously during a registration burst. In this case, we simply assume that a user’s certificate probability will decline in inverse proportion to *N*. This is represented as a $-log(N)$ term within the exponential expression for *S*.

$S=exp\left[ -\left( \frac{D}{E_{U}} \right)^{\beta}-log\left( N \right) \right]$ (6)

Parameter Fitting for the *βEND* Model

| $C_{j}^{i}$ | The probability that registration of user i to course j produces a certificate. |
| --- | --- |
| $E_{U}^{i}$ | The engagement level of user i. |
| $E_{C}^{j}$ | The engagement coefficient of course j. |
| $D_{j}$ | The difficulty level of course j. |
| $\{R^{i}\}$ | The set of courses registered by user i. |
| $\{Z_{k}^{i}\}$ | The set of courses registered by user i during registration burst k. |
| $N_{k}^{i}$ | The total number of courses registered by user i during registration burst k |
| $C_{S}^{j}$ | The certificate rate for singleton users who registered only course j. |
| Table S2. Summary of notation used for parameter fitting. | |

We calculate $C_{j}^{i}$, the probability that user i obtains a certificate for course j following a certain registration event. This calculation requires values for $E_{U}^{i}$, the engagement level of user i; *D_j_* the difficulty of course j; and $N_{k}^{i}$, the number of courses registered by user i during k, the registration burst that includes course j.

The difficulty score *D_j_* is fit as a free parameter for each course. We also fit *β* as a global Weibull shape parameter. Thus our model contains 92 total free parameters: 91 values of *D* and a single *β*.

From *D_j_* and *β*, we calculate $E_{C}^{j}$, the engagement score for course j. This value is constrained by equation 6. Here we make use the singleton certificate rate, $C_{S}^{j}$, which is defined as the certificate rate for users who registered for course j and no other courses. This allows us to take $N=1$ and arrive at the following relation.

$E_{C}^{j}=\frac{D_{j}}{{-log(C_{S}^{j})}^{\frac{1}{\beta}}}$ (7)

Each user, i, is associated with a user engagement score, $E_{U}^{i}$. User engagement scores are estimated as the sum of the course engagement scores over $\{R^{i}\}$, the set of all courses registered by user i in the dataset.

$E_{U}^{i}=\sum E_{C}^{j}\quad\quad j\in\{R^{i}\}$ (8)

A burst event is one subset of registration events for a particular user that are clustered in time. We collect $\{Z_{k}^{i}\}$, the set of courses registered by user i during their kth registration burst. $N_{k}^{i}$ is the total number of courses registered in this event.

$Z_{k}^{i}\subseteq\{R^{i}\}\quad,\quad N_{k}^{i}=\left| \{Z_{k}^{i}\} \right|$ (9)

Finally, we obtain the certificate probability $C_{j}^{i}$, for the registration to course i by user j.

$C_{j}^{i}=exp\left[ -\left( \frac{D_{j}}{E_{U}^{i}} \right)^{\beta}-log\left( N_{k}^{i} \right) \right]$ (10)

Optimal values for *D* and *β* were obtained with the method of maximum likelihood. The parameter search was conducted using the derivative-free simplex method implemented as the fminsearch function in MATLAB(2).

Derivation and Parameter Optimization for the Logistic Model

A logistic model was constructed as a control and benchmark for the performance of the *βEND* model. Following standard practices, the log-odds of course certification, *L*, were estimated as a linear combination of course difficulty, *D_C_* user engagement, *E_U_*, and burst size, *N*.

$L=D_{C}-E_{U}-\gamma N$ (11)

As with the *βEND* model, we estimate user engagement as the sum of course engagement terms, $E_{C}$, for the set of courses registered by the user during the study period, $\{R^{i}\}$.

$E_{U}^{i}=\sum E_{C}^{j}\quad\quad j\in\{R^{i}\}$ (12)

We then made use of the set of single-registered users to derive *E_C_* as a function of *D_C_* reducing the dimensionality of the model.

$E_{C}^{j}=D_{C}^{j}+\gamma+L_{S}^{j}$ (13)

Where $L_{S}^{j}$ is the log certificate odds of singleton users who registered for course j and no other courses.

Optimal values for *D_C_* and *γ* were generated with the method of maximum likelihood using the fminsearch function of MATLAB. Both the *βEND* model and the logistic model were fit with 92 free parameters.

Regression Results for the *βEND* Model

|  |  |  |  |  | **Certificate rate predicted for** | |  | **Pseudo-R^2^ values** | | | |
| --- | --- | --- | --- | --- | --- | --- | --- | --- | --- | --- | --- |
|  | **Sample**  **size** | **Free**  **Parameters** | **Log**  **likelihood** |  | **Certificate**  **earners** | **Non-**  **earners** |  | **McFadden** | **Cox-**  **Snell** | **Nagelkerke** | **Tjur** |
| ***βEND***  **model** | 1,048,531 | 92 | -1.69 ·10^5^ |  | 16% | 7.1% |  | 0.123 | 0.056 | 0.09 | 0.089 |
| **Logistic**  **model** | 1,048,531 | 92 | -1.71·10^5^ |  | 17.5% | 8.4% |  | 0.109 | 0.05 | 0.08 | 0.092 |
| **Null**  **model** | 1,048,531 | 0 | -1.92·10^5^ |  | 8.4% | 8.4% |  |  |  |  |  |
| Table S3. Key properties and performance of the models used in this study. Both the *βEND* and logistic models fit 92 free parameters. These included 91 parameters describing course difficulty. The *βEND* model assigns one free parameter, *β,* to describe the shape of the Weibull hazard function. The logistic model use one free parameter to weight N, the course burst size. The null model simply uses the global mean certificate rate, 8.4%, as the predicted certificate rate for each registration event. Likelihood comparisons to the null model allowed the derivation of pseudo-R^2^ values for both the *βEND* and logistic models. The *βEND* model produced better predictions by all metrics except Tjur's R^2^. The logistic model systematically overestimates certificate probabilities for both certificate earners and non-earners, producing an overall larger prediction gap that is reflected in Tjur's metric. | | | | | | | | | | | |

| **Best-fit values for log(*D_C_*), course difficulty, for 91 courses:** | | | | | | | | |
| --- | --- | --- | --- | --- | --- | --- | --- | --- |
| -6.4 (0.46) | -2.4 (0.27) | -0.62 (0.15) | -0.077 (0.1) | 0.91 (0.29) | 1.9 (0.24) | 2.9 (0.16) | 3.8 (0.25) | 4.7 (0.28) |
| -4.5 (0.17) | -1.9 (0.24) | -0.61 (0.41) | -0.046 (0.03) | 1 (0.13) | 2 (0.12) | 3 (0.24) | 4 (0.32) | 4.9 (0.34) |
| -4.5 (0.18) | -1.6 (0.19) | -0.58 (0.41) | 0.25 (0.12) | 1.1 (0.2) | 2.2 (0.25) | 3 (0.17) | 4 (1) | 4.9 (0.28) |
| -3.9 (0.18) | -1.5 (0.27) | -0.45 (0.39) | 0.33 (0.18) | 1.1 (0.2) | 2.4 (0.2) | 3.3 (0.1) | 4.2 (0.3) | 5.1 (0.19) |
| -3.8 (0.22) | -1.5 (0.49) | -0.32 (0.21) | 0.46 (0.26) | 1.3 (0.34) | 2.5 (0.52) | 3.4 (0.25) | 4.2 (0.29) | 5.4 (0.2) |
| -3.8 (0.28) | -1.4 (0.19) | -0.32 (0.36) | 0.65 (0.08) | 1.4 (0.13) | 2.5 (0.28) | 3.5 (0.2) | 4.2 (0.25) | 5.5 (0.26) |
| -3.7 (0.22) | -1.4 (0.093) | -0.28 (0.12) | 0.73 (0.12) | 1.7 (0.12) | 2.6 (0.49) | 3.5 (0.14) | 4.4 (0.58) | 5.7 (0.13) |
| -3.5 (0.35) | -1.1 (0.12) | -0.23 (0.22) | 0.75 (0.31) | 1.7 (0.3) | 2.6 (0.21) | 3.5 (0.37) | 4.5 (0.56) | 6.4 (1.2) |
| -2.8 (0.19) | -0.89 (0.24) | -0.23 (0.21) | 0.8 (0.18) | 1.7 (0.25) | 2.6 (2) | 3.5 (0.18) | 4.6 (0.16) | 6.4 (0.13) |
| -2.5 (0.22) | -0.81 (0.27) | -0.11 (0.11) | 0.81 (0.38) | 1.7 (0.18) | 2.7 (0.27) | 3.6 (0.63) | 4.6 (0.78) | 6.6 (0.2) |
|  |  |  |  |  |  |  |  | 6.9 (2.5) |
| **Best-fit value for *β*, Weibull shape parameter:** 0.13 (0.0032) | | | | | | | | |
|  | | | | | | | | |
| Table S4. Maximum likelihood parameters for the *βEND* model. The difficulty values for each of 91 courses are ordered from least to most difficult. Standard deviations (in parentheses) for each parameter value were determined by re-fitting 100 bootstrap samples of 10% of the registration events. | | | | | | | | |

Supplementary References

1. Barabasi AL. The origin of bursts and heavy tails in human dynamics. Nature. 2005;435(7039):207-11.

2. Lagarias JC, Reeds JA, Wright MH, Wright PE. Convergence Properties of the Nelder--Mead Simplex Method in Low Dimensions. 1998;9(1):112-47.
