# Supplementary material for: A survival model for course-course interactions in a Massive Open Online Course platform
Source: PLoS One. 2021 Jan 22;16(1):e0245718. doi: 10.1371/journal.pone.0245718 (PMC7822273; doi:10.1371/journal.pone.0245718)
Supplement: Attachment — Submitted filename: Collected Reviewer Comments.docx [file pone.0245718.s002.docx]

Dear PLoS ONE Editor and Reviewers,

Thanks for taking the time to give us your feedback. We respond to each of your comments below and describe the relevant changes to the manuscript.

## Editorial Comment

Please ensure that your manuscript meets PLOS ONE's style requirements, including those for file naming.

## Response and Revisions

We substantially revised our figure files to match the style and format guidelines.

## Editorial Comment

We note that you have indicated that data from this study are available upon request. PLOS only allows data to be available upon request if there are legal or ethical restrictions on sharing data publicly.

In your revised cover letter, please address the following prompts:

If there are no restrictions, please upload the minimal anonymized data set necessary to replicate your study findings as either Supporting Information files or to a stable, public repository and provide us with the relevant URLs, DOIs, or accession numbers.

## Response and Revisions

The underlying data set is now open-access and available for download (DOI: 10.5281/zenodo.3969240)

## Editorial Comment

Please include captions for your Supporting Information files at the end of your manuscript, and update any in-text citations to match accordingly

## Response and Revisions

Captions for Supporting Information figures and tables are now included at the end of the main manuscript text. In-text citations now refer to specific supporting figures and tables, where appropriate.

## Reviewer's Questions

2. Has the statistical analysis been performed appropriately and rigorously?

Reviewer #1: I Don't Know

Reviewer #2: I Don't Know

## Response and Revisions

Our work develops a novel statistical model in a relatively unexplored area of education research. As such, there are few established best practices to guide us. For example, there are no CONSORT or PRISMA guidelines in this community. We affirm that our methods meet PLoS ONE standard of "sufficient detail that others can reproduce the work."

We have increased the statistical documentation that we provide by adding Supporting Information Tables S3 and S4. These regression tables include best-fit parameter values, Pseudo-R^2^ values and log likelihood values obtained using our model and control models.

## Reviewer's Questions

3. Have the authors made all data underlying the findings in their manuscript fully available?

Reviewer #1: No

Reviewer #2: No

## Response and Revisions

The underlying data set is now open-access and available for download (DOI: 10.5281/zenodo.3969240)

## Reviewer's Questions

4. Is the manuscript presented in an intelligible fashion and written in standard English?

Reviewer #1: No

Reviewer #2: Yes

## Response and Revisions

We affirm that our writing meets a high standard of professional English and is intelligible.

Both reviewers offer suggestions regarding the emphasis and organization of points within our manuscript. We have incorporated many of these changes as described below.

## Reviewer #1

Note on data availability – the link provided for the data goes to the general home page of the FUN MOOC platform; there is no indication of how to access the specific data set.

## Response and Revisions

The underlying data set is now open-access and available for download (DOI: 10.5281/zenodo.3969240)

## Reviewer #1

Note on research ethics – the study uses data from human participants, so it is unclear why the ethics statement field is marked N/A

## Response and Revisions

Our study is non-interventional and uses only data collected by a 3rd party (The FUN platform) that was fully anonymized before being transmitted to us. For that reason the study does not meet the definition of "human subjects research" that would require detailed ethical review.

Although our group is based in France, there is broad international agreement on this definition. For reference we provide this English-language document from the US Department of Health and Human Services, in which our research clearly meets the criteria for exemption

<https://www.hhs.gov/ohrp/regulations-and-policy/decision-charts-2018/index.html>

Our Institutional Review Board is hosted by INSERM.

<https://www.inserm.fr/en/gouvernance-organisation/comite-evaluation-ethique>

We have presented our manuscript to Christine Dosquet, the chairwoman of this board, and she has informally agreed that it does not meet the criteria for human subjects research.

Following the standard procedure for our institution, we will submit the revised version of our manuscript to this board prior to publication to obtain official ethical approval. Because this work is not defined as human subjects research, the approval will not require a meeting of the full board and is expected to be completed quickly.

## Reviewer #1

This paper presents some interesting and novel findings relating to the likelihood of MOOC completion, taking into account the effect of users’ overlapping registrations on multiple courses. The suggestion to measure success per burst (of multiple registrations) rather than success per course could be of particular interest to researchers interested in MOOC success factors. In addition the novel approach to analyzing data in this domain highlights some interesting characteristics which have not been evident from previous work, for instance the dichotomy between some courses where users tend to do better in the context of multiple registrations and others where the opposite occurs.

## Reviewer #1

However, I found the presentation of this paper very confusing and therefore difficult to follow. The paper follows an unconventional ordering, with method reported at the end of the paper. I would normally expect to see the following order: introduction, method (data collection, analysis method), results and discussion.

## Response and Revisions

We have greatly expanded the methods section of the paper to include more detail about our data collection and analysis. A reader who seeks out the methods section first will now be able understand the key technical details of our data set and model.

We didn't opt to change the order of the sections to make the methods follow the introduction. This is partially because we come from a field in which the methods conventionally follow the discussion section. We also think that this ordering will be easier to understand for our intended interdisciplinary audience. In particular, we are hoping to reach education researchers with a limited statistics background.

Several of our key findings are either directly actionable for MOOC developers or describe appealing intuitions about how MOOC students learn. For example, we offer education researchers the insight that student drop-out is "aging-like". We hope that that metaphor will draw the interest of this audience and draw them out of their discipline and into the world of statistics. If we put highly technical statistical information at the beginning of the manuscript, these researchers might conclude it was not relevant for them.

## Reviewer #1

In addition I feel that the decisions regarding which material to include under supplementary material and which to foreground in the paper could be reconsidered. Many potentially interesting findings are included under supplementary material, without clear signposting in the main results section, and then appear summarized in the discussion without an indication of where they have come from.

## Response and Revisions

We took this comment to heart and moved a significant portion of our findings from the supplementary material to the main text. Fig 2 now describes bursting registration dynamics and their effect on expected certificate rates. Each panel of this figure was previously provided as supplementary info.

These results are also described in a new subsection of the results entitled "Burst size and burst number show opposing effects on certificate rates" (Page 5, Line 114). The manuscript does read more clearly with the concept of registration bursts moved to the foreground.

The notion of registration bursts are now highlighted with a short paragraph in the introduction, setting reader expectations regarding the central role of bursts in our model.

"The framework of this study is a statistical model for the probability that a given registration event will produce a certificate. Central to this model is the effect of registration bursts, in which users register for multiple courses within a short period of time. We present a simple mathematical relationship between burst size and expected certificate rate." (Page 2, Line 40)

## Response and Revisions

The results reported in the main paper focus mainly on the extent to which the model fits the data, but this is also confusing with much of detail on parameters included in the supplementary material and the meaning of some of findings left quite unclear (e.g. what does it mean in figure 2A to report certification rates at 10-2?).

## Response

We modified fig 2A to express certification rates as a percentage rather than a fraction. It is indeed clearer this way and more consistent with our other figures.

## Reviewer #1

In general the relationship between model parameters and measurable variables could be made much clearer; for example is user engagement something you can measure in reality or purely a theoretical construct here. Also the role of the model in the work could be clarified further by explaining where novel findings about behavior only become observable by using the model.

## Response and Revisions

We have expanded the methods section of the main text to include more details about the model and the parameters used. The section "Characteristics of and Inputs to the *βEND* Model" (Page 12, Line 283) describes each of the parameters input to the model and their theoretical interpretation (e.g. course difficulty).

We have added a paragraph to the discussion section in order to highlight the relationship between our model parameters and other assessments of users and courses.

"A complete understanding of the factors that promote effective web-based learning will require major new programs in education research. Our model quantifies user engagement and course difficulty but does not describe how those properties manifest in the real world. Does user engagement reflect intelligence, motivation, free time or something else? What makes one course difficult and another easy? Interdisciplinary efforts are called for to connect model-derived statistical insights with detailed descriptions of user psychology and course design. Such work may reveal strategies to enhance user engagement and reduce course difficulty." (Pare 11, Line 249)

## Reviewer #1

For me, the evidence regarding the fit of the model compared to regression is less interesting than the novel understanding of behavioural patterns that emerge from the analysis (and form the focus of much of the discussion) and may be more appropriate for the supplementary section.

## Response and Revisions

We agree that our model is most interesting when it is used to understand real users and courses. Figure 5 is presented as a proof-of-concept to how how model-derived parameters can reveal interesting patterns in user behavior by age. Integration of the model with other demographic data represents the next step in this line of research.

Before that can happen, we need to convince people 1) That a model can provide mechanistic insights beyond simple classification and 2) That the *βEND* model is a well founded theoretical approach to understanding online learners.

We see this paper as the "due diligence" phase of describing a new quantitative theory of learning. For that reason we wanted to demonstrate the qualitative agreement of the *βEND* model with the structure of the underlying data and to show that it outperforms naïve approaches like simple logistic regression.

In short the purpose of fig 3 and our presentation of the model's fitting performance is a kind of "eat your vegetables" moment that we hope will convince future researchers to consider *βEND* or other aging-like models as the preferred tool for a learning dataset.

## Reviewer #2

Thank you for the opportunity to review your paper. I think it's definitely an enjoyable read and an interesting topic you are addressing.

The paper is understandably written and original. Therefore, I would like to address certain key points where I think this manuscript could be improved before publication. These keypoints are thoughts and tips for analyzing the data in a different way, which might lead to somewhat different conclusions. I would like to see these points addressed or answered.

## Reviewer #2

(1) You mention that only 8.1% of all registrations lead to a certificate. I think this number of events is very low, especially when facing such a big dataset. Therefore, I would like you to consider fitting a cure model. This class of survival models takes into account that a proportion of your sample is 'not susceptible' for the event-of-interest (i.e., certification). I guess that some courses might be way too difficult, so barely anyone will get a certification OR some users (cfr. engagement) are roamers who subscribe without any intention of completing a course (e.g., for revision or whatever intent), so they will never complete a course. A third possibility is maybe the combination too (hardest courses can only be completed when the user is sufficiently engaged).

I think these analysis tools might yield much more robust results. Anyway, I would like to hear your thoughts on it and address this issue/possibility in the paper.

## Response and Revisions

We agree with your central insight about user behavior. Most users are probably 'not susceptible' to obtain a certification because they never intended to obtain one. The question is how can we capture this intuition in a quantitative model?

The constraint of a cure model is that it defines a fixed proportion of the population as 'not susceptible.' This, in turn, fixes an upper limit on the certification rate for any course no matter how easy.

We would like our model to be scalable across MOOC platforms, including platforms that offer courses with widely ranging difficulty levels. In principle this could include courses that are arbitrarily easy. Imagine a tutorial course in which users only have to answer a single question to obtain a certificate. If any courses on the platform have very high certificate rates, it will not be possible to set a global non-susceptible population.

A more detailed approach would define a non-susceptible population specifically for each course. Naturally this requires many more parameters. The *βEND* model in fact does something similar to this. Because the certification rate drops very quickly with *D_C_* for small *D_C_*, a large number of users drop out long before *D_C_* is reached for most courses (Fig R1). There are relatively few courses in our data set where *D_C_* is small enough that a cure model would be a poor approximation.

|  | **Fig R1. Predicted certificate rates in the *βEND* model as a function of course difficulty.** We used the best-fit value of 0.13 for *β* and *E_U_* = 1 as a typical user engagement level. Typical course difficulties in our data set ranged from 20-50. For these difficulty values, the *βEND* model gives "cure-like" behavior because a large cohort of users drop out long before the *D_C_* value is approached. However, some courses in our data set were very easy, with *D_C_* values less than 1. In this regime, drop-out behavior may not be cure-like. |
| --- | --- |

Your point raises another interesting question: "How non-susceptible are the non-susceptible users?" In other words, how easy does a course need to be before the majority of users would make some effort towards completing it. Are the very early drop-outs willing to click even one button? Or do they drop out after putting literally zero effort. Our data set doesn't answer this question but we would like to see it in future work.

## Reviewer #2

(2) It remains unclear to me, how you addressed the course-course interaction in the paper. It is not entirely clear which model you used (a fixed effects model with course dummies I suppose). This made it hard to understand how you link your results to course-interactions.

## Response and Revisions

Course-course interactions are captured in our model because we use all of the courses registered by a given user to predict that user's engagement level, *E_U_*. A user who has registered for a very high *D_C_* course (e.g. quantum physics) and a very low *D_C_* course (e.g. basic arithmetic) is more likely to obtain a certificate than a user who registered only for the easy course. This is reflected in the model by simply summing the *D_C_* values for all courses that a user has registered and using that for their *E_U_* value.

This is an important part of our model and, we agree, somewhat counterintuitive. So we have expanded the places in the manuscript where the idea is reiterated. We have greatly expanded the Methods section and include this text under the section "Characteristics of and Inputs to the *βEND* Model"

"The *E_U_* term for each registration event is calculated as the sum of *D_C_* terms for all courses registered by that user during the study period." (Page 12, Line 295).

This notion is also described in Fig 1, in line 46 of the Introduction and line 64 of the Results.

I would, again, like to propose an alternative: use a cross-level frailty model. Your course-user observations can be seen as having a cross-level structure which you can take into account by considering your observation to be nested in both users and courses. Hence, you get more specific estimates on user-level effects (User Engagement) and course-level effects (Difficulty). These models immediately allow to infer on the correlation structure of random effects.

## Response and Revisions

We don't see an easy way to adapt our data for use in a cross-level frailty model. The key difference between our data and the data that you would use for a conventional survival analysis is that we don't have access to normal survival time measurements.

We wish we had a measurement of "drop out time" for each individual student. This would be exactly analogous to "time of death" in survival analysis. If the data was structured in this way we could bin the data by time, calculate the relative hazard rates for each cohort at each time point, then model those relative rates with a Cox-like model with cross-level terms for whatever parameters you like.

But instead we have only a single time point for each registration. Did the user's survival time exceed the course difficulty or not? Without more time-resolved data, a simple cross-level analysis would look like the logistic regression that we perform as a control.

You raise the important point that more time-resolved data would unlock more detailed survival analysis. We discuss this further in your comment about the Cox model below.

## Reviewer #2

(3) Why do you need cross-validation? Why is this used on your data and what are the results? Did you use it to assess standard errors? To train the model in a Machine-Learning-like way? I don't get why this is necessary for your model?

## Response and Revisions

You're right, it is not particularly necessary. Cross-validation is, in our experience, "the kind of thing that reviewers ask for". It is a nonspecific form of due diligence that can catch various kinds of error. Over-fit can sneak into any complex model and cross-validation helps to avoid it.

We are not trying to make any strong claims about the performance of our model as a predictive classifier. We are content to demonstrate that our model agrees with retrospective data and provides new ways to quantify and understand user behavior.

## Reviewer #2

Next, I would like to address certain keypoints which will make the paper easier to understand for a broader audience.

## Reviewer #2

(4) Could you please elaborate a little on the BEND model you are using. I had never heard about this model and, correct me if I am wrong, I think not many other researchers will recognize the model immediately. I think I would be great to know a little about the model and its relation to more common survival models, possibly even non-parametric survival models such as the Cox model.

In this, I would also like to see a short discussion on how well the model fits. It has been found multiple times that misspecifying a baseline hazard might lead to biased results (which could be avoided by juxtaposing your model with a Cox model, which leaves the baseline hazard unspecified).

## Response and Revisions

The *βEND* model is novel to this work and, as far as we know, nothing similar has been developed elsewhere. Unlike the Cox model and other popular statistical hazard models, it is not designed to be generalizable. Instead, it reflects our specific mechanistic hypotheses regarding how users behave in online courses. For example:

- Users divide their attention evenly between courses registered in a burst. Thus, predicted certificate rates go exactly as 1/N.
- Student drop out is caused by the "weakest link" of many possible causes, resulting in Weibull aging dynamics.
- A student's engagement level is reflected by the courses that they register, allowing E_U_ to be predicted as the sum of D_C_.

So the *βEND* model is "theory rich" and intentionally parametric. Where a logistic model might find that N correlates with certification rates, we say that relationship has an exact mathematical form. Where a Cox model is robust to any nonparametric form of aging, we constrain ourselves to the Weibull function because we think that drop-out follows "weakest link" dynamics and not other dynamics.

Of course, we could be wrong. You make a very good point about the Cox model in particular and the potential of introducing biases by fixing a specific baseline hazard function.

As we discussed above, we don't have access to well defined user drop-out times. For that reason, we can't say if these drop-out times follow a Weibull distribution as we claim. If we are wrong and drop-out times are non-parametric, then Cox-like models will be the right tool for future analyses.

We have added a paragraph to the discussion that highlights this:

"The *βEND* model formally connects MOOC certificate rates to systems reliability theory. As user data becomes more detailed and time-resolved, survival analysis will help us to understand why and when users lose interest in learning online. While we find the FUNCERT data set is well described by Weibull-like aging dynamics, MOOC structures will continue to evolve. Future work should explore new and existing hazard functions to find models that generalize across platforms while revealing common patterns in user behavior over time." (Page 11, Line 261)

## Reviewer #2

(5) I would like a regression table equivalent to judge which covariates are in your model. It is not clear to me whether the discussion of the results comes from one integrated model (where you display marginal effects) or a series of split-sample (post-hoc) models. Personally, I think the second option may suffer from some severe drawbacks (e.g., omitted variable bias, confounding effects, ...).

## Response and Revisions

We added tables S3 and S4 as Supporting Information. These tables give the best-fit model parameters and various metrics regarding their performance like pseudo-R^2^ values.

All of our results come from a single model and a single set of best-fit parameters.

## Reviewer #2

(6) What is the rationale of the registration bursts? Why don't you left-truncate the data, so every observation line is a registration spell?

You can take the number of simultaneous spells into account as a covariate (or allow differences in baseline?).

## Response and Revisions

Bursts are often found in online user behavior (SI Reference 1). We wanted to characterize registration bursts in detail to show that they follow similar dynamics to other forms of bursting. In that way, we can be confident that similar dynamics will occur on other MOOC platforms.

Left truncating the data would probably work fine. A key conclusion of our analysis is that registration bursts are mostly well defined and unambiguous. So any way of grouping them should work.

We like agglomerative clustering because it seems to behave better in certain edge cases. Imagine a user who registered for 3 courses in 1 day, each registration 4 hours apart. If we use agglomerative clustering with a cutoff of 8 hours, those three events are grouped together. If we left truncate with a threshold of 8 hours, the third event would be separated from the other two.

But it would be hard to compare the performance of different clustering strategies given that they should all perform well.

## Reviewer #2

Lastly, I will make some minor comments:
- please add a reference to your claim that extreme value theory regards the minimum failure time of n components to be on average Weibull distributed (line 74)

- please check 'weird' line breaks (e.g., lines 98 and 125), it looks like text has disappeared there.

## Response and Revisions

We have added a citation to Fisher & Tippett, 1928. The type-setting errors have been corrected.

## Reviewer #2

Hence, I will conclude my review of your paper.
I hope to have incited the authors to think about alternative analyses and their potential benefits to the data at hand. I hope you will engage in a conversation and review of your article along these lines.

I have made myself known, so please feel free to contact me if at any point I was not clear.

## Response and Revisions

We spoke to Dr. Hans Tierens in the course of preparing these revisions. It was our first experience working with a self-identified reviewer and we found the direct feedback to be extremely valuable. Particularly when discussing complex and subtle ideas, there is just no substitute for interactive conversation.

Thanks to all for a productive review process!
