# Supplementary material for: A survival model for course-course interactions in a Massive Open Online Course platform
Source: PLoS One. 2021 Jan 22;16(1):e0245718. doi: 10.1371/journal.pone.0245718 (PMC7822273; doi:10.1371/journal.pone.0245718)
Supplement: Attachment — Submitted filename: Collected Reviewer Comments v2.docx [file pone.0245718.s003.docx]

Dear PLoS ONE Editor and Reviewers,

Thanks for the positive feedback and constructive criticism. For brevity we are omit the reviewers' many kind words and focus only on outstanding issues below.

## Reviewer #1

Reviewer #1: The paper reads much more clearly now and is now more accessible for educational researchers. Issues around data access and research ethics have also been satisfactorily addressed in the revisions.

## Reviewer #2

Firstly, I think that the authors have formulated some nice and clear answers to the reviewer comments. However, I think that much of these explanations deserve a place in the actual manuscript. More specifically, I am referring to:

1) the elaboration on ‘cured participants’ (i.e., participants who did never subscribe for courses with the aim of obtaining a certificate, and are, thus, insusceptible for the event of obtaining one). I think that this would greatly improve on ways forward in this research domain, as well as a clear guideline on what data is required to continue along this line.

(On a side note, I don’t think that ‘cure’ can be reliably inferred when looking at certification rates across course difficulty (DC). Course difficulty is inferred by the model itself and does not (always) reflect a real time basis. I would suggest restricting Try to make this real time.
Besides this comment, I think that ‘small cure proportions’ is no valid argument to dismiss the reliability of a cure model. In some respect, cure rate models without time varying covariates are really alike a zero-inflated Poisson model and, basically, any selection model or model with truncated dependent variables... In this sense, ‘cure’ proportions of “only” 7% were already found to improve statistical inference (see Blevins et al. 2015 in Organizational Research Methods and Antonakis et al. 2014 in Leadership Quarterly).

## Response and Revisions

We have added a paragraph to the discussion to highlight these points and included a reference to the Blevins paper. Cure models will have an important place in future drop-out research. We present a challenge to the community to determine if low-engagement users are really "cured" or whether they retain some possibility of "infection."

"Survival models may help to reveal the causes and consequences of the low-engagement registrations that frustrate many MOOC providers(17). One possibility is that a certain proportion of registered users have a strictly zero probability of obtaining a certificate. Cure models in survival analysis(18) or zero-inflated models in organizational research(19) are appropriate for describing such a fully disinterested population. The *βEND* model, in contrast, allows user engagement levels to be low but non-zero and suggests that small increases in engagement will increase raise rates among low-engagement users. Future studies could resolve these competing hypotheses by identifying user behaviors consistent with strictly zero or non-zero engagement. Such studies will require high temporal resolution in the period immediately following registration, when survival models indicate a fast transition from persistence to drop-out." (Page 11, Line 248)

## Reviewer #2

2) You claim that the βEND model is “theory rich”, to which I fully agree. However, please bring this more forward in the manuscript. Preferrably, when explaining all the different theory-driven building blocks of the model. I believe that this evidence of a theory-rich model the current manuscript, referring to the added paragraph, is a little short and does not delve too deeply into the ‘theory’ as compared to your response to the reviewers.

In the same light, a theory-rich model necessitates a part of the discussion on how each of your theory-driven choices in developing the building blocks could be ‘untrue’ and how researchers can use their data (which was currently unavailable in your dataset) to improve and test some of these theoretical assumptions/boundaries of the model (for example, baseline hazard, functional form, assumed distributions) as well as elaborate and extend your model using additional data (e.g., course difficulty and user engagement might not be ‘just functions of each other’ but may depend on a set of covariates).

## Response and Revisions

We have added a paragraph to the discussion that reminds readers of the assumptions behind our theory and the places where future work could make improvements. As in our response to the comment above, the motivation is to challenge the community with opportunity to refine the model.

"The *βEND* model formally connects MOOC certificate rates to systems reliability theory. Within this domain a rich set of mathematical tools will support more detailed analysis of the dynamics of dropout. We have demonstrated a parametric approach featuring several strong theoretical assumptions. First, we propose that the mechanisms of dropout follow weakest-link scaling and therefore adopt a Weibull distribution in time. Second, we consider certificate rate probabilities to be divided equally among multiple registrations. While this approximation agrees with our data it is likely that users are making motivated and non-random decisions about how to focus their attention. Finally we assume the independence of user engagement and course difficulty when these two parameters likely feature complex interactions and covariates. As data sets become richer and more time-resolved it may become possible to develop parametric or non-parametric reliability models that are richer or more generalizeable. Future work will also need to consider MOOC formats that continuously evolve to meet the needs of users and providers(17)." (Page 13, Line 321)

## Reviewer #2

3) I have one additional comment on the clustering process to group subscriptions into bursts. You claim that you wanted to make the model as scalable as possible, which means that researchers in the field can (easily) use your model to gain more insights in their data. However, in what way and to what extent is the clustering process scalable? I think that the rationale of ‘bursts are often found in online user behavior’ is hardly an argument for scalability. If anything, please reflect on this issue in the discussion section of your manuscript.

## Response

Agglomerative clustering is scaleable in the sense that it is a simple technique that is implemented in most data science software packages.

There are even simpler approaches, for example simple truncation, but these can give counterintuitive results in certain edge cases. Consider 3 registration events 7 hours apart. Truncation with a cutoff of 8 hours would group two of these events together and exclude the third arbitrarily. Agglomerative clustering would group all 3 together.

Our results show that registration events are well clustered in time. Therefore any choice of clustering algorithm would likely give similar performance. If we highlight our choice of clustering algorithm in the discussion, there is a risk that the reader will believe is it a more complex and innovative method than it really is. We don't think our approach to clustering is a central contribution and would rather not devote more space.

## Reviewer #2

4) I still don’t think that cross-validation is necessary in this paper and I fear it takes away focus from the core (contribution) of this model. If it is added because of ‘something reviewers will ask for’, I would like to clearly state its purpose in the manuscript and how it may help researchers in their inference.

## Response and Revisions

We have modified the methods section to specify that bootstrap replicates were used to determine parameter confidence intervals. Cross-validation is not discussed elsewhere in the manuscript, so we think that readers will not misunderstand that our model was intended to be a predictive classifier.

"In total we fit 92 free parameters: 91 values of DC for the 91 certificate-awarding courses and a single value of *β*, the Weibull shape parameter. Maximum likelihood estimation of the free parameter values was performed using the fminsearch function of MATLAB. Parameter confidence intervals were determined using 100 bootstrap replicates in which 330,000 users were randomly assigned to a training groups and the remainder to a test group. All reported statistics of the performance of the model are on users of the test group. A complete regression table including best-fit parameter values is provided as Supporting Information Table S4." (Page 4, Line 91)

## Reviewer #2

Lastly, I would like to reinforce a comment of my colleague reviewer (reviewer 1) and counter a response of the authors. I really think that it is advisable to rework the ordering of the sections in the manuscript. I agree that it might be usual in your field to let the methods follow the discussion, but I think for a big portion of the audience of PLOS ONE this ordering seems weird and highly unconventional. I think that readers with a limited statistical background will just skip formulaic expressions and read through them searching for some theoretical foundations of the building blocks of the model. It makes a non-statistical reader also more aware of how the model works, before you present the results of your study. Following the more conventional order (in our field at least); literature, method, results, discussion, readers will better understand the results, where they came from and how to interpret them. I would suggest to write more for the target audience of your journal.

## Response and Revisions

OK we concede the point! Because both reviewers prefer the methods-first organization, we have moved the methods section to the beginning of the manuscript following the introduction.
